# Mock Plant Communities and a Large Mammal Case Study Reveal ITS2 Primer Bias Against Graminoids

**DOI:** 10.1002/ece3.72102

**Published:** 2025-09-07

**Authors:** Mary Sadyrova, Emily Martin, Philip Ramsey, Lorinda Bullington

**Affiliations:** ^1^ MPG Ranch Florence Montana USA; ^2^ Department of Ecosystem and Conservation Sciences University of Montana Missoula Montana USA

**Keywords:** diet barcoding, Illumina MiSeq, molecular diet analysis, plant mock community, ungulate

## Abstract

DNA fecal metabarcoding has revolutionized the field of herbivore diet analyses, offering deeper insight into plant‐herbivore interactions and more reliable ecological inferences. However, due to PCR amplification bias, primer selection has a major impact on the validity of these inferences and insights. Using two pooling approaches on four mock communities and a case study examining diets of four large mammalian herbivores (LMH), we evaluated the efficacy of two primer pairs targeting the internal transcribed spacer 2 (ITS2) region: the widely used ITS‐S2F/ITS4 pair and the UniPlant F/R pair, designed specifically for DNA metabarcoding. Both primer pairs consistently underrepresented graminoids, where > 40% of graminoid species did not amplify in vitro. However, the UniPlant F/R primer pair more accurately amplified mock plant communities, whereas the ITS‐S2F/ITS4 pair underestimated graminoid relative abundance by at least twofold more than UniPlant F/R primers. Furthermore, in the LMH case study, UniPlant F/R primers identified graminoids as the dominant plant group for at least one LMH, indicating diet niche partitioning, while ITS‐S2F/ITS4 primers largely failed to amplify graminoid DNA, potentially overestimating LMH diet overlap. Our findings underscore the importance of incorporating mock community analyses into DNA metabarcoding protocols to identify and mitigate primer bias, thereby enhancing the accuracy of ecological conclusions and supporting more effective conservation and management decisions.

## Graphical abstract

DNA fecal metabarcoding has greatly advanced herbivore diet analysis, but primer selection plays an important role in mitigating PCR amplification bias, affecting ecological inferences. In this study, using plant mock communities and a large mammalian herbivore case study, we evaluated two primer pairs targeting the ITS2 region. Both primers underrepresented graminoids, but the UniPlant F/R pair more accurately amplified plant communities and detected diet niche partitioning in herbivores. Our findings highlight the need for mock community analyses for identifying and addressing primer bias, thereby enhancing the accuracy of ecological conclusions and supporting more effective conservation and management decisions.

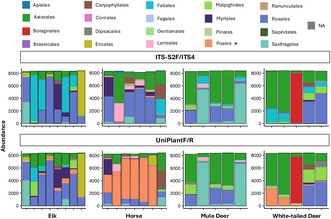

## 1 Introduction

Over the past decade, DNA metabarcoding has enabled more comprehensive assessments of biodiversity and community composition for many types of environmental samples. Progress has been particularly advantageous in diet studies (Moorhouse‐Gann et al. 2018), allowing deeper insight and analysis of complex herbivore‐plant interactions (Da Silva et al. 2019; Moorhouse‐Gann et al. 2022; Pitteloud et al. 2023). Diet and body size of large mammalian herbivores (LMH) have been key factors in determining their impact on ecosystems (Pringle et al. 2023). Considerable effort has been devoted to diet reconstruction, as it is by far the more difficult of the two factors to measure (Chesson 2000; Pansu et al. 2022; Simberloff and Dayan 1991). Older microhistological approaches for diet analysis often underrepresented forbs due to their softer cell walls and higher digestibility compared to graminoids, shrub buds, and tree bark (King and Schoenecker 2019). Newer methods based on DNA fecal metabarcoding give a fuller picture of LMH diets and have greatly enhanced our understanding of fundamental ecological concepts, such as competition, niche partitioning, and complementarity (Kartzinel et al. 2015; Pansu et al. 2022). However, despite recent advancements, accurate diet reconstruction of free‐living LMH still faces challenges.

A universal plant DNA barcode remains elusive due to the challenge of balancing broad applicability with high species‐level resolution, both essential for accurate diet reconstruction. This challenge is compounded by inter‐ and intraspecific variability among plant groups, along with PCR amplification biases (Moinard et al. 2023; Rieseberg et al. 2006). An ideal barcode marker should offer species‐level discrimination, broad applicability, and ample DNA amplification and sequencing (CBOL Plant Working Group et al. 2009; Taberlet et al. 2007). However, designing universal primers that target conserved flanking regions and sufficiently variable internal regions requires extensive in silico and in vitro validation. In 2011, the China Plant Barcode of Life (BOL) group tested the nuclear ribosomal internal transcribed spacer (ITS), along with multiple other plastid regions (China Plant BOL Group et al. 2011). While ITS demonstrated less universality, performing better in angiosperms than gymnosperms, it displayed greater discriminatory power, enabling classification down to genus and species. Acknowledging the tradeoffs, ITS was subsequently recommended for consideration as a core barcode for plants, offering a more cost‐effective alternative to the earlier two‐locus approach (Braukmann et al. 2017; CBOL Plant Working Group et al. 2009; China Plant BOL Group et al. 2011; Hollingsworth et al. 2011).

The ITS2 region, a shorter fragment of the full ITS marker, has become a popular choice for classifying vascular plants. Its shorter length (225–581 bp) (Cheng et al. 2016; Espinosa Prieto et al. 2024) simplifies amplification and sequencing, while maintaining relatively high taxonomic resolution for distinguishing closely related taxa (Braukmann et al. 2017; Chase et al. 2005; Chen et al. 2010; Kolter and Gemeinholzer 2021b; Yao et al. 2010). Additionally, the availability of reference databases, such as UNITE (Abarenkov et al. 2024), has contributed to the barcode's popularity. Early concerns about paralogous copies, fungal contamination, and issues amplifying and sequencing bulk samples appear less challenging than previously thought, with the benefits often outweighing these limitations (China Plant BOL Group et al. 2011; Hollingsworth et al. 2011; Kolter and Gemeinholzer 2021a; Moorhouse‐Gann et al. 2018). The numerous advantages of this marker have established it as a preferred choice for analyzing environmental samples.

Despite its growing popularity and broad applications, biases in ITS2 primer performance remain equivocal. Primer‐associated PCR amplification biases can affect dietary reconstruction by distorting taxonomic resolution and relative abundance estimates, thereby obscuring our understanding of interactions between herbivores and their food resources. Biases can often arise from both natural and technical sources (Angly et al. 2014; Cheng et al. 2016; Espinosa Prieto et al. 2024; Freeland 2017; Mallona et al. 2011; Nichols et al. 2018; Stadhouders et al. 2010). Perhaps the biggest contributor to taxonomic abundance distortion stems from primer‐template mismatches as a result of poor primer design and level of universality (Deagle et al. 2014; Espinosa Prieto et al. 2024; Liu et al. 2023; Piñol et al. 2019; Stadhouders et al. 2010). Furthermore, in working with environmental DNA (eDNA), especially in fecal metabarcoding, bias can be compounded by unavoidable natural processes, such as plant digestibility and DNA degradation (Ando et al. 2020; Elbrecht and Leese 2015; Krehenwinkel et al. 2017; Moinard et al. 2023). Since accurate taxonomic abundance estimates are not always straightforward, primer selection becomes increasingly important as it can alleviate or exacerbate these biases, independently.

We evaluated the efficacy of two primer pairs commonly used to target the plant ITS2 barcode region. First, ITS‐S2F/ITS4 (~363 bp), a widely used primer pair in eDNA plant metabarcoding studies (Chen et al. 2010; Espinosa Prieto et al. 2024; White et al. 1990). Second, UniPlant F/R (187–387 bp), a primer pair designed with greater universality for higher taxonomic resolution in herbivory analyses (Moorhouse‐Gann et al. 2018). We based our selection of these pairs on their high prevalence in the literature and on multiple published comprehensive in silico surveys of plant ITS2 primers (Cheng et al. 2016; Espinosa Prieto et al. 2024; Kolter and Gemeinholzer 2021a). However, because in silico analyses are not always indicative of primer performance on actual environmental samples (e.g., Kolter and Gemeinholzer 2021a; Moorhouse‐Gann et al. 2018), we expand upon previous studies by comparing the two chosen primer pairs on individual plant samples, plant mock communities, and via a herbivore case study to thoroughly assess primer performance. Furthermore, we employed four mock community composition types and two pooling approaches to better isolate PCR bias.

**FIGURE 1:**
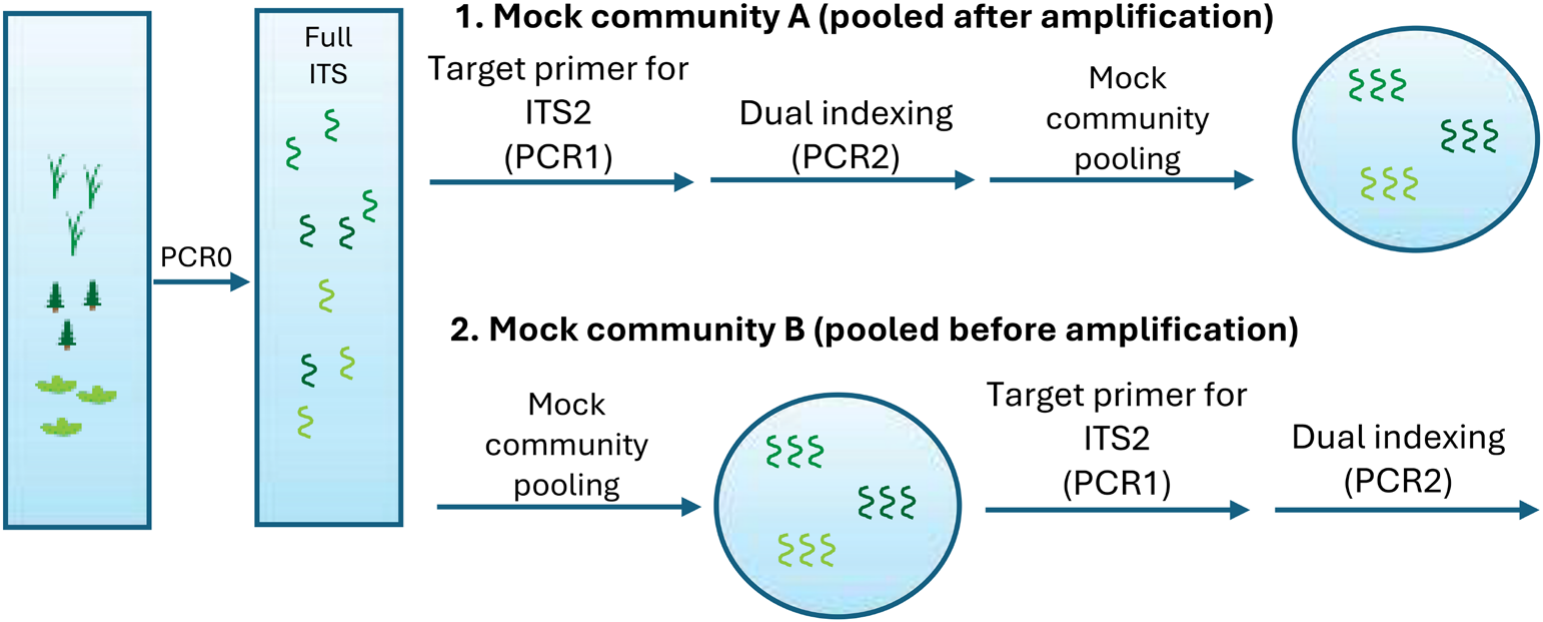
Schematic depicting the mock community experimental design for (1) Mock community A (MC‐A) where plant DNA was pooled *after* primer amplification and dual indexing and for (2) Mock community B (MC‐B) where DNA was pooled *before* primer amplification and dual indexing. The full internal transcribed spacer region was amplified (nested PCR) for all individual plant species (PCR0) before pooling or primer amplification in both MC‐A and MC‐B to reduce fungal or other non‐target contamination and improve accuracy of target‐species quantification. The blue circles with DNA strands depict when mock communities were pooled during the workflow.

To address potential biases associated with each primer pair during PCR amplification, we evaluated two pooling approaches (Figure 1) in four mock community composition types (Table 1). All mock communities were comprised of three main plant life form groups: graminoids, forbs, and trees/shrubs. The four composition types included (1) an equal community, consisting of even representation of all species, (2) grass dominant, (3) forb dominant, or (4) tree and shrub dominant. The purpose of the different composition types was to assess if communities could be accurately differentiated based on plant relative abundance. In the first pooling approach, DNA from all plant specimens was amplified independently with each primer pair, followed by dual‐indexing for sample identification, and lastly pooled into communities with known DNA concentrations. This is hereafter referred to as Mock Community A (MC‐A, pooled *after* primer amplification), representing communities with “expected” concentrations of DNA. In the second approach, we pooled plant DNA in known concentrations *before* amplifying communities with each primer pair, followed by dual‐indexing. This is hereafter referred to as Mock Community B (MC‐B, pooled *before* primer amplification). This dual‐pooling approach allowed us to compare the extent of potential bias introduced by each primer pair compared to the expected community pooled immediately before Illumina sequencing. Overall, we assessed 16 treatments: 2 primer pairs × 2 pooling approaches × 4 community composition types.

**TABLE 1:** Structure of plant mock community composition types (equal, forb dominant, grass dominant, tree/shrub dominant). Gray boxes indicate species with 3× representation within their respective dominant mock communities.

Scientific name	Life form	Equal	Forb dominant	Grass dominant	Tree/shrub dominant
*Agastache urticifolia*	Forb	1×	3×	1×	1×
*Bassia scoparia*	Forb	1×	3×	1×	1×
*Campanula rotundifolia*	Forb	1×	3×	1×	1×
*Erigeron speciosus*	Forb	1×	3×	1×	1×
*Erodium cicutarium*	Forb	1×	3×	1×	1×
*Helianthus annuus*	Forb	1×	3×	1×	1×
*Medicago sativa*	Forb	1×	3×	1×	1×
*Myosotis laxa*	Forb	1×	3×	1×	1×
*Rudbeckia hirta*	Forb	1×	3×	1×	1×
*Tragopogon dubius*	Forb	1×	3×	1×	1×
*Urtica dioica*	Forb	1×	3×	1×	1×
*Verbascum thapsus*	Forb	1×	3×	1×	1×
*Veronica anagallis‐aquatica*	Forb	1×	3×	1×	1×
*Agropyron cristatum*	Graminoid	1×	1×	3×	1×
*Bromus tectorum*	Graminoid	1×	1×	3×	1×
*Carex geyeri*	Graminoid	1×	1×	3×	1×
*Elymus cinereus*	Graminoid	1×	1×	3×	1×
*Festuca campestris*	Graminoid	1×	1×	3×	1×
*Festuca idahoensis*	Graminoid	1×	1×	3×	1×
*Panicum capillare*	Graminoid	1×	1×	3×	1×
*Phleum pratense*	Graminoid	1×	1×	3×	1×
*Trisetum canescens*	Graminoid	1×	1×	3×	1×
*Berberis repens*	Shrub	1×	1×	1×	3×
*Cornus sericea*	Shrub	1×	1×	1×	3×
*Rosa gymnocarpa*	Shrub	1×	1×	1×	3×
*Rosa woodsii*	Shrub	1×	1×	1×	3×
*Pinus ponderosa*	Tree	1×	1×	1×	3×
*Populus tremuloides*	Tree	1×	1×	1×	3×
*Pseudotsuga menziesii*	Tree	1×	1×	1×	3×

Each primer set was also applied to fecal samples collected from coexisting large mammalian herbivores. Samples were collected from elk (*Cervus canadensis nelsoni*), mule deer ( *Odocoileus hemionus* ), white‐tailed deer ( *Odocoileus virginianus* ), and feral horses ( *Equus caballus* ) in the Bitterroot Valley, Montana, USA (46°40′48′′ N, 114°1′40′′ W). Feral horse populations are a contentious issue for managers worldwide due to concerns over their impact on ecosystem processes and competition with native ungulates (Scasta et al. 2016; Stoner et al. 2021). Mule deer populations across the western United States have experienced declines over recent decades, while elk populations have increased (Stewart et al. 2010). These trends raise concerns that competition for forage contributes to the declines (Bergman et al. 2015). We evaluated how primer selection could influence interpretations of diet in terms of competition versus niche complementarity.

Overall, due to variability in nucleotide mismatch and specific amplicon lengths targeted, we expect that the two primer pairs would show consistent differences in amplification of individual plant species, mock communities, and environmental DNA. Because biases in mock communities should represent potential biases that may occur with environmental samples, we expect that patterns observed in the LMH case study would reflect biases evident in MC‐B. However, due to natural degradation that inevitably occurs with environmental DNA, these biases could become either more or less pronounced in fecal samples.

## 2 Methods

### 2.1 Plant Specimen Collection and DNA Extraction

To construct mock communities, we collected 29 plant specimens (including 9 graminoids, 13 forbs, and 7 trees/shrubs) common to the Bitterroot Valley, Montana, USA, with some being widespread across North America and globally. Species were selected to represent a variety of plant life forms and combinations of taxa observed to occur in local LMH diets (Bullington et al., in prep) and with a generally high relative abundance on the landscape. Plant foliar tissue was collected while avoiding inclusion of seeds or flowers. Samples were first freeze‐dried using a Labconco Freezone benchtop freeze‐dry system for 24 h (Labconco Corp. Kansas City, MO, USA), then macerated with a 1600 MiniG tissue homogenizer and cell lyser (Spex SamplePrep, Metuchen, NJ, USA), and finally stored at −20°C prior to DNA extraction. Genomic DNA was extracted from 15 mg of ground foliar tissue using either a Qiagen DNeasy Plant Pro Kit (Cat# 69206) or DNeasy PowerPlant Pro Kit (Cat# 13400) (Qiagen, Germantown, MD, USA), following manufacturer instructions. Extraction blanks were included to monitor laboratory contamination.

### 2.2 Mock Community DNA Amplification and Library Prep

#### 2.2.1 Nested PCR Amplification of Individual Specimens

For mock communities, we performed a nested PCR by first amplifying the entire internal transcribed spacer (ITS) region of each plant species individually using the plant‐specific forward primer ITS‐p5 (Cheng et al. 2016) and the general eukaryotic reverse primer ITS4 (White et al. 1990) to obtain amplicons of ~800 nucleotides containing the downstream ITS2 target region (see Table A1 in Appendix A for primer sequences). From hereon we will refer to this PCR product as PCR0 (Figure 1). This allowed for subsequent quantification of pure target plant DNA for each individual specimen, and it reduced DNA contamination from non‐target organisms (e.g., endophytic fungal or bacterial communities that associate with plant tissues). Amplification was carried out in 37.5 μL reaction volumes containing 3 μL DNA template, 80 ng/μL BSA (New England BioLabs, Ipswich, MA, USA), and 0.2 pmol of each primer in 1× GoTaq Green Master Mix (Promega, Madison, WI, USA). Control blanks were included with each PCR run. Reactions were performed using a SimpliAmp Thermal Cycler (Thermo Fisher Scientific, Waltham, MA, USA). For a detailed description of the nested PCR settings used, see Appendix B section “Nested PCR Conditions”. To confirm the presence of our target amplicon and absence of multiple PCR products, reactions were analyzed by 1.5% agarose gel electrophoresis. Amplification blanks were included in each round of PCR, and no contamination was detected. Amplicons were then purified using AMPure XP beads (Beckman Coulter Genomics, Chaska, MN, USA), quantified with a Qubit 2.0 fluorometer (Invitrogen, Waltham, MA, USA), and standardized to a final concentration of 0.3 ng/μL.

#### 2.2.2 MC‐A and MC‐B Construction and PCR

We employed two approaches for pooling mock communities to address potential biases in the PCR amplification process: (1) quantifying and pooling individual species after PCR amplification and dual‐indexing (MC‐A), and (2) quantifying and pooling individual species before PCR amplification and dual‐indexing (MC‐B). To construct MC‐A, PCR0 products from individual plant specimens were amplified in PCR1, targeting the ITS2 region using either the NEXTERA tagged primer pair NEX1‐UniPlantF/NEX2‐UniPlantR or NEX1‐ITS2‐S2F/NEX2‐ITS4 (Table A1). Dual‐indices (NEXTERA i5 or i7 indices) were added to each individual plant species in PCR2, followed by amplicon purification using AMPure XP beads and quantification with a Qubit 2.0 fluorometer. Purified, dual‐indexed amplicons were then combined to create the mock community compositions described above with the desired final DNA amount of 50 ng for 1× samples and 150 ng for 3× samples, represented by two duplicate reactions each. For MC‐B, PCR0 product representing each specimen was combined into each of the four mock community compositions as described above, with 1.5 ng for 1× samples and 4.5 ng for 3× samples, represented by three duplicate reactions each. These samples were then prepared for Illumina sequencing using a two‐step PCR process with each primer pair. Briefly, PCR1 included 12.5 μL reaction volumes containing 1 μL of mock community template, 80 ng/μL BSA (New England BioLabs, Ipswich, MA, USA), and 0.2 pmol of each primer in 1× GoTaq Green Master Mix (Promega, Madison, WI, USA). For PCR2, primer complexes consisted of Illumina adapters, Nextera tags (NEX1 or NEX2) and 8‐bp Nextera i5 or i7 barcodes (Illumina Inc., San Diego, CA, USA). PCR2 was carried out in 25 μL reaction volumes containing 1 μL of PCR1 product as template, 40 ng/μL BSA (New England BioLabs, Ipswich, MA, USA), and 0.2 pmol of each primer in 1× GoTaq Green Master Mix (Promega, Madison, WI, USA). For a detailed description of the PCR1 and PCR2 settings used, please see Appendix B section “MC‐A and MC‐B PCR Conditions”. Amplicons were purified and quantified using AMPure XP beads and a Qubit 2.0 fluorometer. Multiple extraction blanks and PCR blanks for each step were sequenced. No contamination was detected.

#### 2.2.3 Individual Specimen PCR Amplification Test

For in vitro amplification tests of each individual plant specimen, DNA was amplified using each primer pair. Illumina library prep and purification was performed as described above, with each reaction representing a single plant taxon. For Illumina sequencing, all MC‐A, MC‐B, and individual plant samples were added to the final library in equimolar concentrations. Sequencing was performed at the University of Montana Genomics Core (UMGC, Missoula, MT, USA). Amplicon libraries were sequenced using a MiSeq v2 kit (500 cycles) on an Illumina MiSeq sequencing platform (Illumina Inc., San Diego, CA, USA).

### 2.3 Reference Plant Database Construction

Thorough evaluation of primers for environmental samples requires a comprehensive reference library of local specimens. We collected specimen material from 195 of the most abundant vascular plants occurring at and around MPG Ranch (46°40′48′′ N, 114°1′40′′ W; www.mpgranch.com). This included all species represented in the mock communities. MPG Ranch is a privately owned property dedicated to conservation and ecological research, located in the Bitterroot Valley of western Montana. This area is characterized by semi‐arid grasslands that support a diverse mix of native and introduced plant species. DNA extractions were performed as described above. We amplified the ITS2 region using the primer pair ITS2‐S2F and ITS4. We then used paired‐end Sanger sequencing of each amplified product (Eurofins Genomics, KY, USA) to obtain reference sequences for each specimen. Ambiguous bases were trimmed from the ends of forward and reverse reads and consensus sequences were generated using BioEdit with default parameters (Alzohairy 2011). All sequences that did not overlap were removed, resulting in a final local plant library representing 161 locally abundant plant species. We then obtained sequences representing the ITS2 region of an additional 26 species from GenBank, for a final library representing 187 local plant species.

### 2.4 Mammalian Herbivore Case Study

In July 2019, we collected fecal samples from elk (*Cervus canadensis nelsoni*; *n* = 8), mule deer ( *Odocoileus hemionus* ; *n* = 5), white‐tailed deer ( *Odocoileus virginianus* ; *n* = 5), and feral horses ( *Equus caballus* ; *n* = 7). All sampling occurred on MPG Ranch, requiring no permits, and in adherence with the local state and federal regulation and guidelines for non‐invasive fecal sampling, posing no risk, harm, or disturbance to local wildlife. The vegetation, with species lists and phenological information, has been previously described (Durham et al. 2017). All fecal samples were collected immediately after deposition to ensure freshness. To minimize external contamination and maximize the representation of ingested plant material, we sampled from multiple interior locations within each pellet for DNA extraction. Samples were freeze‐dried using a Labconco Freezone benchtop freeze‐dry system for 72 h (Labconco Corp. Kansas City, MO, USA) and macerated with a 1600 MiniG tissue homogenizer and cell lyser (Spex SamplePrep, Metuchen, NJ, USA). Genomic DNA was extracted from 75 mg of ground fecal sample using a Qiagen Dneasy PowerSoil Pro (Cat# 47016) (Qiagen, Germantown, MD, USA). Template was diluted 1:2× before using the same two‐step amplification as used for both mock communities described above. All extractions and PCR runs included quality control blanks, and no contamination was observed. Amplicons were pooled and purified using AMPure XP beads and quantified with a Qubit 2.0 fluorometer. Amplicon libraries were sequenced using the same process described above.

### 2.5 Bioinformatics

**FIGURE C1:**
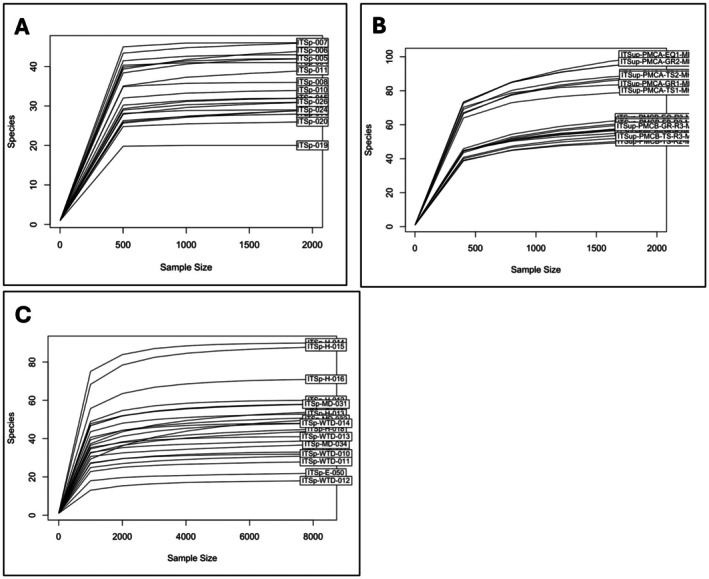
Rarefaction curves are shown for samples from mock communities (MC‐A and MC‐B) amplified with (A) ITS‐S2F/ITS4 and (B) UniPlantF/R primers, rarefied to 2000 reads per sample, and (C) LMH samples amplified with both primer pairs, rarefied to 8400 reads per sample. Depths were selected based on rarefaction curves indicating sufficient depth for retaining all samples and recovering community diversity.

Raw sequence reads were obtained from the Illumina MiSeq platform and processed with Quantitative Insights into Microbial Ecology 2 (QIIME2) v. 2023.7 (Bolyen et al. 2019). Forward and reverse reads were first demultiplexed, followed by primer trimming using the cutadapt trim‐paired plugin (Martin 2011). Next, reads were denoised using the dada2 denoise‐paired plugin (Callahan et al. 2016) by correcting for sequence errors and identifying biological amplicon sequence variants (ASVs). All reads were quality trimmed, maintaining an average base quality score of 30 or greater. Taxonomy was first assigned to representative sequences using the local plant library and the classify‐consensus‐vsearch plugin (Rognes et al. 2016). Taxonomic assignments were based on at least 99% identity and 80% coverage. For sequences unassigned to the local database, subsequent assignment was performed using QIIME2's classify‐sklearn method with a naïve Bayes classifier (Pedregosa et al. 2011) trained on a database containing both our local reference library and the UNITE all eukaryote database v. 9.0 (Abarenkov et al. 2024). All sequences that could not be assigned to kingdom Viridiplantae using this method were removed from further analyses. Samples representing mock communities were rarefied to a depth of 2000 reads per sample for even comparison, and samples for the LMH comparison were rarefied to a depth of 8400 reads in order to retain the most reads without losing samples (see Figure C1 in Appendix C). However, assessment of plant species detection was performed on unrarefied data to maximize detection potential. All samples passed quality filtering and were retained after rarefaction.

### 2.6 Statistics

All statistics were performed using R version 4.4.1 (R Core Team 2021). To determine if plant functional group abundances differed between MC‐A and MC‐B, for each primer pair and each mock community abundance type, we assessed log‐fold changes between each plant functional group. Using the “Procrustes” function in the package Vegan 2.7‐1 (Oksanen et al. 2024) we aligned MC‐B datasets with synthetically generated data tables representing each plant community abundance type rarefied to the same number of sequences as the observed mock communities. To perform permutation tests to assess the statistical significance of each alignment, we used the “protest” function. This analysis tests whether the observed similarity between two datasets is greater than expected by chance.

To quantify differences in diet composition among herbivore species in the LMH study, we performed Permutational Analysis of Variance (permANOVA) using the Adonis2 function also in the package Vegan. All permANOVA was performed on Bray–Curtis distances of Hellinger transformed, rarefied sequence abundances using 1000 permutations. To visualize differences in diet composition among herbivores, we performed Nonmetric Multidimensional Scaling (NMDS) using the metaMDS function in the package Vegan and plotted results using the package ggplot2 v.3.5.2 (Wickham 2016).

## 3 Results

### 3.1 Detection of Mock Community Taxa

For in vitro analyses using single‐plant amplification and sequencing, the UniPlant F/R primer pair successfully amplified 96.6% of plants at the genus level and 75.9% of plants at the species level (Table 2). For the ITS‐S2F/ITS4 primer pair, amplification success was 93.1% and 72.4% at the genus and species levels, respectively. Amplification success differed by one plant specimen, *Festuca campestris* , that ITS‐S2F/ITS4 primers failed to amplify altogether. Notably, ITS‐S2F/ITS4 primers successfully identified *Festuca idahoensis* to the genus level. For MC‐B, where plant DNA was pooled before primer amplification allowing for potentially greater effects of primer bias, fewer genera and species were detected by both primer pairs. In MC‐B samples, the UniPlant F/R primers detected four more genera than ITS‐SF2/ITS4 primers, including *Agastache, Myosotis, Urtica*, and *Phleum*, and seven more species. In graminoids specifically, UniPlant F/R detected two more species than ITS‐SF2/ITS4: *Agropyron cristatum* and *Festuca campestris* . Neither primer pair detected the two gymnosperm species, *Pinus ponderosa* and *Pseudotsuga menziesii* , in MC‐B samples.

**TABLE 2:** Representation of plant genera and species detection by both primer pairs, comparing in vitro detection, at genus and species levels of independently amplified plant specimens, with detection in mock communities pooled before target amplification (MC‐B).

Mock community			ITS‐S2F/ITS4				UniPlant F/R			
Genus	Species	Life form	In vitro detection		MC‐B only		In vitro detection		MC‐B only	
			Genus	Species	Genus	Species	Genus	Species	Genus	Species
*Agastache*	*urticifolia*	Forb	✓	✓	✗	✗	✓	✓	✓	✓
*Bassia*	*scoparia*	Forb	✓	✓	✓	✓	✓	✓	✓	✓
*Campanula*	*rotundifolia*	Forb	✓	✓	✓	✗	✓	✓	✓	✓
*Erigeron*	*speciosus*	Forb	✓	✗	✗	✗	✓	✗	✗	✗
*Erodium*	*cicutarium*	Forb	✓	✓	✓	✓	✓	✓	✓	✓
*Helianthus*	*annuus*	Forb	✓	✓	✓	✓	✓	✓	✓	✓
*Medicago*	*sativa*	Forb	✓	✓	✓	✓	✓	✓	✓	✓
*Myosotis*	*laxa*	Forb	✓	✓	✗	✗	✓	✓	✓	✓
*Rudbeckia*	*hirta*	Forb	✓	✓	✓	✓	✓	✓	✓	✓
*Tragopogon*	*dubius*	Forb	✓	✓	✓	✓	✓	✓	✓	✓
*Urtica*	*dioica*	Forb	✓	✓	✗	✗	✓	✓	✓	✓
*Verbascum*	*thapsus*	Forb	✓	✓	✓	✗	✓	✓	✓	✓
*Veronica*	*anagallis‐aquatica*	Forb	✓	✗	✓	✗	✓	✗	✓	✗
*Agropyron*	*cristatum*	Graminoid	✓	✓	✓	✗	✓	✓	✓	✓
*Bromus*	*tectorum*	Graminoid	✓	✓	✓	✓	✓	✓	✓	✓
*Carex*	*geyeri*	Graminoid	✓	✓	✓	✓	✓	✓	✓	✓
*Elymus*	*cinereus*	Graminoid	✓	✗	✓	✗	✓	✗	✓	✗
*Festuca*	*campestris*	Graminoid	✗	✗	✓	✗	✓	✓	✓	✓
*Festuca*	*idahoensis*	Graminoid	✓	✗	✓	✗	✓	✗	✓	✗
*Panicum*	*capillare*	Graminoid	✓	✓	✓	✓	✓	✓	✓	✓
*Phleum*	*pratense*	Graminoid	✓	✗	✗	✗	✓	✗	✓	✗
*Trisetum*	*canescens*	Graminoid	✗	✗	✗	✗	✗	✗	✗	✗
*Berberis*	*repens*	Shrub	✓	✓	✓	✓	✓	✓	✓	✓
*Cornus*	*sericea*	Shrub	✓	✓	✗	✗	✓	✓	✗	✗
*Rosa*	*gymnocarpa*	Shrub	✓	✓	✓	✓	✓	✓	✓	✓
*Rosa*	*woodsii*	Shrub	✓	✓	✓	✗	✓	✓	✓	✗
*Pinus*	*ponderosa*	Tree	✓	✓	✗	✗	✓	✓	✗	✗
*Populus*	*tremuloides*	Tree	✓	✗	✓	✗	✓	✗	✓	✗
*Pseudotsuga*	*menziesii*	Tree	✓	✓	✗	✗	✓	✓	✗	✗

### 3.2 Differences in Plant Functional Group Relative Abundances

**FIGURE 2:**
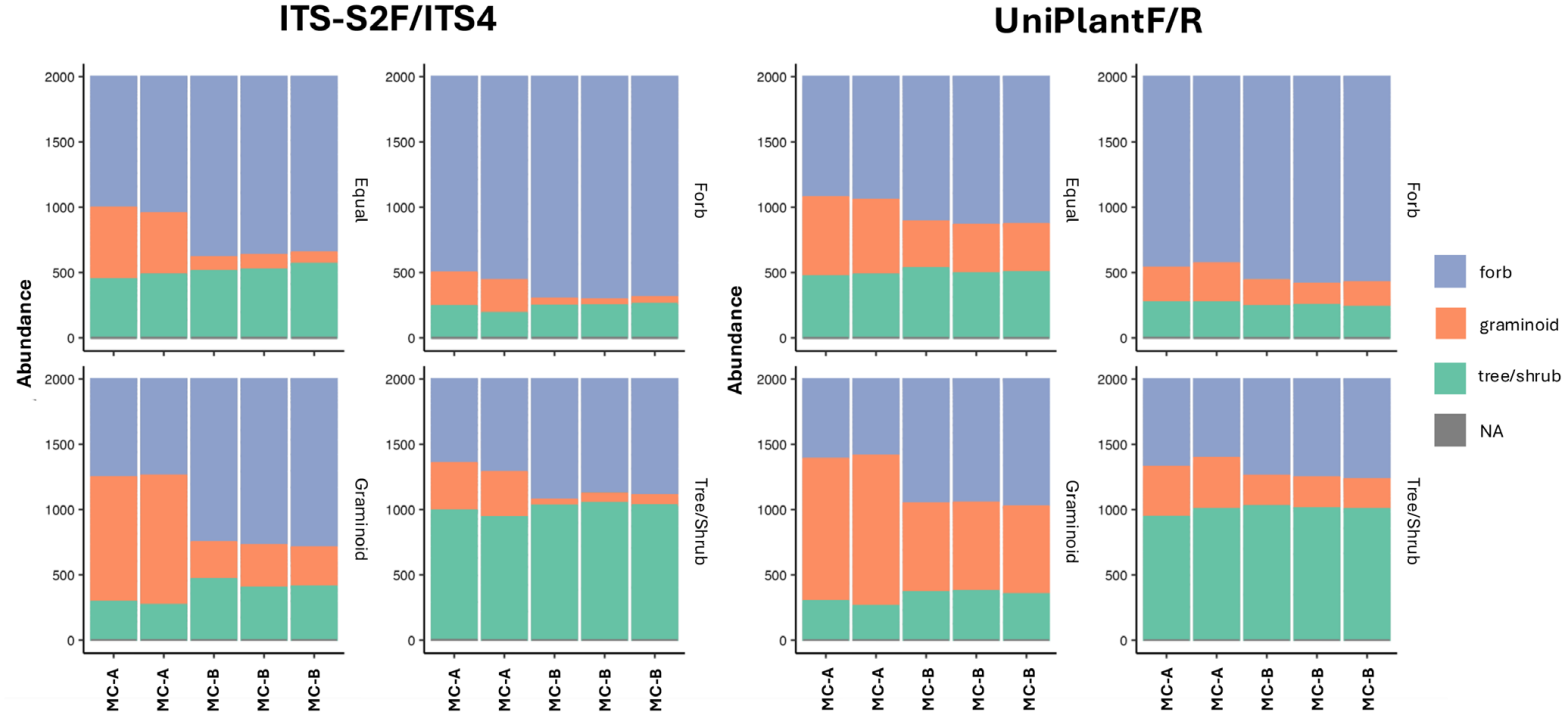
Stacked bar plot depicting expected plant functional group relative sequence abundances (graminoid, forb, tree/shrub) in plant mock community A (MC‐A) compared to observed abundances in plant mock community B (MC‐B), as amplified by ITS‐S2F/ITS4 and UniPlant F/R primer pairs. Bars represent MC‐A and MC‐B PCR replicates. Relative sequence abundances of plant functional groups for each community abundance type are represented on the *y*‐axis.

Assessing the relative abundances of plant functional groups after amplification revealed significant underrepresentation of graminoids in MC‐B compared to MC‐A by both primer pairs in all mock community abundance types (Figure 2). However, based on the log‐fold change values associated with differences between MC‐A (“expected” community, with less opportunity for primer bias) and MC‐B abundances (“observed” community, with primer bias analogous to amplification of environmental samples), the bias against graminoids was at least 2× greater with ITS‐S2F/ITS4 primers compared to UniPlant F/R primers. This was true in all but the graminoid‐dominant community, where both primer sets underrepresented graminoids to a similar degree (Table 3). For ITS‐S2F/ITS4, both forb and tree/shrub functional groups were also overrepresented in all but the tree/shrub‐dominant communities. In contrast, for UniPlant F/R primers, forb and tree/shrub abundances did not differ between MC‐A and MC‐B in either graminoid or tree/shrub‐dominant communities, and forbs did not differ in abundance between MC‐A and MC‐B in forb‐dominant communities.

**TABLE 3:** Results of changes in plant functional groups (graminoids, forbs, trees and shrubs) in plant mock community B (MC‐B) compared to mock community A (MC‐A). Results indicate differences in relative abundances of groups represented by the log‐fold change (lfc).

Mock community abundance type	Functional group	ITS‐S2f/ITS4	UniPlant F/R
		lfc	lfc
Even	Graminoid	−1.56	−0.27
	Forb	0.35	0.4
	Tree/shrub	0.19	0.28
Graminoid dominant	Graminoid	−0.70	−0.78
	Forb	1.00	0.18
	Tree/shrub	0.88	−0.01
Forb dominant	Graminoid	−1.21	−0.55
	Forb	0.51	−0.04
	Tree/shrub	0.57	−0.24
Tree/shrub dominant	Graminoid	−1.81	−0.59
	Forb	0.19	0.09
	Tree/shrub	−0.01	−0.04

**FIGURE 3:**
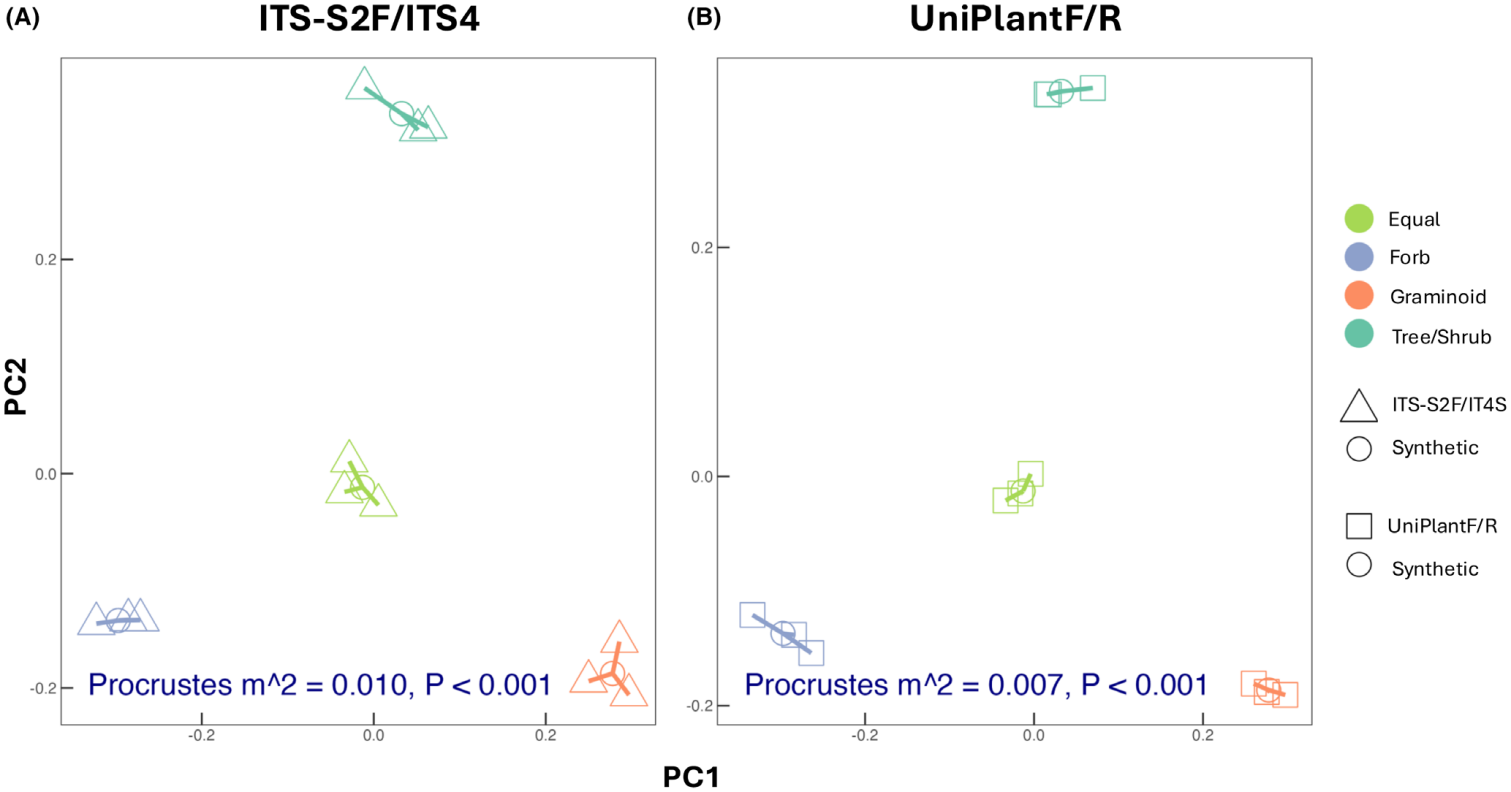
Procrustes analysis comparing simulated expected (circles) and observed (triangles or squares) mock plant community composition before amplification (MC‐B) with plant specific primers (A) ITS‐S2F/ITS4 or (B) UniPlant F/R. Plant communities were standardized to 2000 sequences per sample.

Both primer pairs significantly differentiated between mock community abundance types (i.e., even, forb dominant, graminoid dominant, & tree/shrub dominant) with minimal variation between replicates (Figure 3). However, UniPlant F/R primers showed slightly less variation from the simulated dataset representing each abundance type than ITS‐S2F/ITS4. This was assessed using the “protest” function where a lower m^2^ value associated with UniPlant primers indicated a greater similarity between simulated and observed MC‐B configurations.

### 3.3 Large Mammal Case Study

**FIGURE 4:**
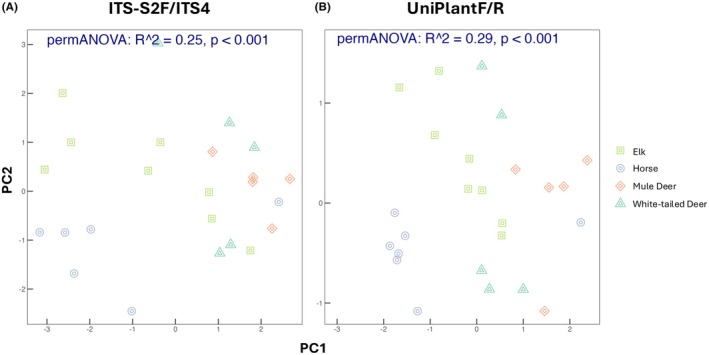
Principle coordinate analyses (PCoA) representing vascular plant diets of elk, horses, mule deer, and white‐tailed deer using ITS‐S2F/ITS4 (A) or UniPlant F/R primers (B). Points represent fecal samples collected in western Montana, USA in July of 2019. Stress was < 0.10.

**FIGURE 5:**
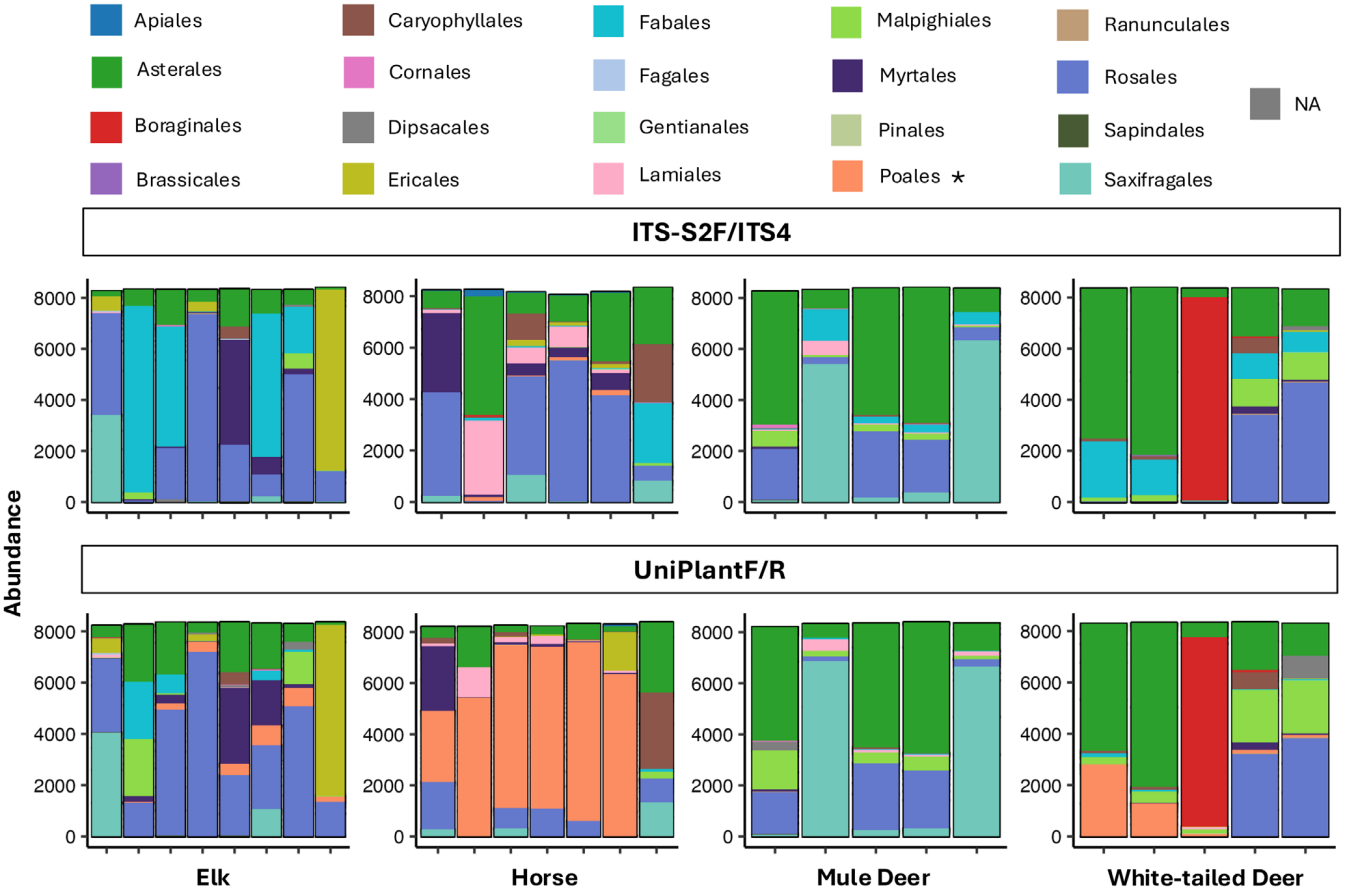
Bar chart of relative sequence abundances of plant orders (*y*‐axis) in the diets of elk, horses, mule deer, and white‐tailed deer using ITS‐S2F/ITS4 (top row) and UniPlant F/R (bottom row) primers to amplify plant DNA extracted from fecal pellets collected in western Montana, USA in July of 2019. Bars represent individual fecal piles. Asterisk (*) indicates order containing all graminoids.

The permANOVA indicated differences in diet among the four co‐occurring large mammalian herbivores when using each primer pair (Figure 4), with no significant differences in dispersion among herbivores for either primer set (*p* > 0.05). However, herbivore species explained slightly more variation in diet when using UniPlant F/R primers. When comparing the relative abundance of plant orders within each mammal diet, UniPlant F/R primers amplified primarily graminoids belonging to the order Poales in horse diets, whereas Poales were rarely detected in horse diets using ITS‐S2F/ITS4 primers (Figure 5). In herbivore samples where Poales were not detected, diet composition appeared largely similar between primer sets.

## 4 Discussion

While diet metabarcoding of the ITS2 plant barcoding region offers many advantages over traditional methods, this study demonstrates that primer choice affects the accuracy of herbivore diet reconstruction, where graminoids are a major component. Inherent natural biases and introduced technical biases can influence taxonomic resolution and DNA abundance estimates (Fonseca 2018; Moinard et al. 2023; Nichols et al. 2018). Natural biases, such as those that stem from different digestion rates, may be unavoidable; however, work remains to be done in this field to address technical bias. Despite primers successfully amplifying most plant species in vitro, we found that the ITS‐S2F/ITS4 primers consistently underrepresented graminoid abundance in mock plant communities twofold compared to UniPlant primers, a pattern seemingly amplified in fecal samples in the LMH case study. In contrast, UniPlant F/R primers produced a closer match between observed (MC‐B) and expected plant communities (MC‐A). UniPlant F/R primers were also more effective in amplifying taxa at both the genus and species levels, particularly for graminoids. Nevertheless, inherent biases in bulk‐sampled mock communities (MC‐B) were still apparent with both primer pairs.

In selecting ITS2 primers, one must consider the trade‐offs between species‐level resolution, even coverage across taxa, and the specific goals of the study. While some primers provide fine‐scale resolution for dicots, they may underrepresent other taxonomic groups. The ITS‐S2F/ITS4 primers are thus far the most widely used universal ITS2 primer pair (Espinosa Prieto et al. 2024). Over the past decade, researchers have applied these primers in herbivore diet analyses (Quin et al. 2023; Thomassen et al. 2023; Yamamoto and Uchida 2018), plant surveys (Fahner et al. 2016; Frøslev et al. 2017), palynology analyses (Sickel et al. 2015; Swenson and Gemeinholzer 2021), and plant‐pollinator interaction studies (Bänsch et al. 2020; Khansaritoreh et al. 2020; Lim et al. 2018; Wilson et al. 2021). Previous herbivore diet studies have frequently reported low graminoid abundance. For example, Thomassen et al. (2023) noted that graminoids were underrepresented in horse diets, which they attributed to an abundance of forbs in the study area. Yamamoto and Uchida (2018) inferred that a grasshopper species avoided a grass from the Poaceae family, even though it was the most abundant and second tallest species in the study area. However, considering this primer pairs' bias toward grasses, the grasshopper species may have had a higher proclivity toward the most abundant plant species. Similarly, Quin et al. (2023) reported low graminoid abundance in sambar deer diets, contrasting previous studies using morphological methods (Padmalal et al. 2003; Stafford 1997). Our findings demonstrate clear evidence of primer bias against graminoids, suggesting they may be more abundant in some herbivore diets than previously recognized, and highlighting the need for further investigation into their role in diet reconstruction.

In silico analyses by Kolter and Gemeinholzer (2021a) independently evaluated ITS primers for mismatch rates within Spermatophyta families, providing further insight into observed primer bias. Both ITS‐S2F and ITS4 primers performed poorly among Poales, with mismatches for both Poaceae and Cyperaceae families. Alternatively, the UniPlant reverse primer performed slightly better, and the UniPlant forward primer performed best overall, having the lowest mismatch rate among Poales families. All mismatches among Poales were a result of a single base pair nucleotide replacement (either A or T with a C or G). Furthermore, in silico analyses of the UniPlant primer pair by Moorhouse‐Gann et al. (2018) revealed a higher mismatch rate for monocot grasses (Poales), relative to other plant orders, and particularly for Cyperaceae family due to poor reverse primer fit. However, their in vitro results showed a much higher amplification success compared to in silico (99% and 88% respectively), and a 100% amplification success of Poales. In our study, while the UniPlant pair performed comparatively better than the ITS‐S2F/ITS4 pair, it still underestimated grass abundance in mock communities. In addition, nearly 17% and 38% of plants failed to amplify at the genus and species levels respectively in MC‐B (Table 2). These inconsistencies highlight the importance of primer selection and the need for in vitro and in vivo validation, as well as the development of alternative methods and further refinement of existing primers. Nevertheless, our results, showing the close relationship between observed and expected communities, suggest that UniPlant F/R primers will improve the estimation of herbivore diet composition and dietary niche partitioning, making it the preferred primer pair choice, particularly for studies focused on grass‐dominated diets.

Both primer pairs evaluated in this study performed similarly in samples appearing to lack graminoids, effectively detecting broad differences in plant community compositions. However, notable discrepancies emerged in herbivore diets containing graminoids, indicating that biases observed in mock communities may be amplified in fecal samples. In the LMH case study, UniPlant F/R primers identified graminoids as the dominant plant group in horse diets, while ITS‐S2F/ITS4 primers largely failed to amplify graminoid DNA, obscuring true diet diversity. Such biases may overestimate dietary overlap among species, emphasizing forbs over grasses. In just using ITS‐S2F/ITS4 primers, feral horses might be viewed as competing directly with native cervids for food. However, if horses primarily consume grasses, their grazing could reduce grass cover and promote forb growth, potentially facilitating resource partitioning and supporting plant community diversity (Arsenault and Owen‐Smith 2002; Augustine and Springer 2013). We sampled over a period of only 1 month in our case study; however, for a more comprehensive and accurate picture of how these species interact and how resource partitioning varies seasonally, it would require at least a full year of sampling (Stewart et al. 2010). Nevertheless, both primer pairs successfully differentiated between herbivore species even over this narrow range of time.

Diet is a key factor in determining the impact of large mammalian herbivores on ecosystems through their influence on plant demography, biomass, species composition, and nutrient cycling (Pringle et al. 2023). For instance, if exotic deer focus on forbs rather than grasses, their grazing could disproportionately affect rare plant species, increasing conservation concerns (Quin et al. 2023). Similarly, identifying whether feral horses compete with or complement native ungulates requires accurate dietary data. Our case study illustrates how biases in primer selection may misrepresent niche differentiation, and ultimately our interpretation of herbivore ecology. At broad taxonomic levels, species may appear to compete for resources, but finer‐scale analyses often reveal facilitative interactions. Overlapping diets can mask specialization, where species balance trade‐offs between forage quantity and quality, reducing direct competition and fostering coexistence. This misrepresentation risks flawed ecological interpretations which could lead to poor management decisions. Without accurate data to understand the intricacies of herbivore diets, managers may implement strategies that fail to address specific ecological concerns and the impacts of the species in question.

Despite their utility, mock communities remain underused in DNA metabarcoding, and standardizing their inclusion could improve the reliability and reproducibility of ecological inferences (Moorhouse‐Gann et al. 2018; Nichols et al. 2018). In our work, the mock community approach allowed for evaluation and comparison of two primer pairs in how they perform in respect to each other and across a variety of mock community compositions (equal, grass dominant, forb dominant, or tree/shrub dominant). Primer validation is vital for increasing the accuracy of herbivore diet barcoding data, which is key for identifying patterns in trophic interactions (Meyer et al. 2020), competitive overlaps between species (Pitteloud et al. 2023), or simply in determining whether a species' dietary needs are being met (Castle et al. 2020). The goal of our study was not to advocate in favor of any one primer pair more broadly, but rather to demonstrate the implications primer bias can have on the accuracy of results, and in the context of herbivore diet studies, the downstream ecological inferences. An emerging alternative approach, such as camera collar video, may illuminate certain DNA metabarcoding biases by enabling species‐level taxonomic identification and providing a relatively reliable estimate of how frequently specific taxa are consumed by the herbivore. However, this approach is not without its own limitations. Collecting and processing video data is expensive, time‐consuming, and is highly susceptible to human bias and error. Additionally, video availability is biased toward a certain time frame, and can be highly invasive or simply impossible in some systems (Johnson et al. 2025; Béland et al. 2023; Newmaster et al. 2013; Thompson et al. 2012). Therefore, future work should prioritize developing alternative barcode markers to expand taxonomic coverage and precision, thereby improving dietary reconstruction. These advancements have broad applications for understanding herbivore impacts, niche partitioning, resource availability, and in addressing conservation challenges.

## 5 Conclusion

A DNA fecal metabarcoding approach provides a more complete picture of herbivore diets and improves our understanding of various ecological processes as opposed to older methods, such as direct observation and microhistology (Kartzinel et al. 2015; Pansu et al. 2022). Though certain natural biases might be unavoidable, researchers should do everything possible to address and mitigate the effects of technical biases. We recommend that mock community validation be integrated as a standard step in diet metabarcoding workflows. Our study highlights the importance of primer selection and associated mock community analyses at the local scale to help expose some of these underlying biases (Timpano et al. 2020). Advancements in the field of fecal DNA metabarcoding could lead to more accurate dietary data, which underpins our understanding of ecological dynamics.

## Author Contributions

**Mary Sadyrova:** writing – original draft (lead), writing – review and editing (lead). **Emily Martin:** conceptualization (equal), methodology (lead), writing – review and editing (supporting). **Philip Ramsey:** writing – original draft (supporting). **Lorinda Bullington:** conceptualization (equal), formal analysis (lead), writing – original draft (supporting).

## Conflicts of Interest

The authors declare no conflicts of interest.

## Data Availability

Raw sequence reads have been deposited in the SRA (BioProject PRJNA1218679). R code used for figures and analyses has been deposited into Figshare under https://doi.org/10.6084/m9.figshare.29631437.v1.

## Appendix A

**TABLE A1:** Names and sequences of ITS2 plant primers and NEXTERA tags used.

Name	Description	Primer sequence
UniPlantF	Forward primer	TGTGAATTGCARRATYCMG
UniPlantR	Reverse primer	CCCGHYTGAYYTGRGGTCDC
ITS2‐S2F	Forward primer	ATGCGATACTTGGTGTGAAT
ITS‐p5	Forward primer	CCTTATCAYTTAGAGGAAGGAG
ITS4	Reverse primer	TCCTCCGCTTATTGATATGC
NEX1	Forward NEXTERA tag	TCGTCGGCAGCGTCAGATGTGTATAAGAGACAG
NEX2	Reverse NEXTERA tag	GTCTCGTGGGCTCGGAGATGTGTATAAGAGACAG

## Appendix B

### Additional Methods

#### Nested PCR Conditions

For the nested PCR, we amplified the full ITS regions of each plant species individually using plant‐specific forward primer ITS‐p5 (Cheng et al. 2016) and the general eukaryotic reverse primer ITS4 (White et al. 1990). Reactions were performed under the following conditions: initial denaturation for 2 min at 94°C, followed by 35 cycles of 30 s at 94°C, 30 s at 57°C, and 30 s at 72°C, and a final extension for 7 min at 72°C, before storage at 4°C.

#### MC‐A and MC‐B PCR Conditions

The following PCR1 amplification conditions were used for the ITS2‐S2F/ITS4 primer pair: initial denaturation for 2 min at 94°C, followed by 35 cycles of 30 s at 94°C, 30 s at 57°C, and 30 s at 72°C, and a final extension for 7 min at 72°C. Similarly, the following conditions were used for the UniPlantF/R primer pair: initial denaturation for 10 min at 95°C, followed by 40 cycles of 30 s at 95°C, 30 s at 56°C, and 1 min at 72°C, and a final extension for 10 min at 72°C (Moorhouse‐Gann et al. 2018). PCR 2 reactions were performed under the following conditions: 95°C for 1 min; 15 cycles of 95°C for 30 s, 60°C for 30 s, and 68°C for 1 min; and 68°C for 5 min.

## References

[ece372102-bib-0001] Abarenkov, K. , R. H. Nilsson , K.‐H. Larsson , et al. 2024. “The UNITE Database for Molecular Identification and Taxonomic Communication of Fungi and Other Eukaryotes: Sequences, Taxa and Classifications Reconsidered.” Nucleic Acids Research 52, no. D1: D791–D797. 10.1093/nar/gkad1039. 37953409 PMC10767974

[ece372102-bib-0002] Alzohairy, A. 2011. “BioEdit: An Important Software for Molecular Biology.” GERF Bulletin of Biosciences 2: 60–61.

[ece372102-bib-0003] Ando, H. , H. Mukai , T. Komura , T. Dewi , M. Ando , and Y. Isagi . 2020. “Methodological Trends and Perspectives of Animal Dietary Studies by Noninvasive Fecal DNA Metabarcoding.” Environmental DNA 2, no. 4: 391–406. 10.1002/edn3.117.

[ece372102-bib-0004] Angly, F. E. , P. G. Dennis , A. Skarshewski , I. Vanwonterghem , P. Hugenholtz , and G. W. Tyson . 2014. “CopyRighter: A Rapid Tool for Improving the Accuracy of Microbial Community Profiles Through Lineage‐Specific Gene Copy Number Correction.” Microbiome 2, no. 1: 11. 10.1186/2049-2618-2-11. 24708850 PMC4021573

[ece372102-bib-0005] Arsenault, R. , and N. Owen‐Smith . 2002. “Facilitation Versus Competition in Grazing Herbivore Assemblages.” Oikos 97, no. 3: 313–318. 10.1034/j.1600-0706.2002.970301.x.

[ece372102-bib-0006] Augustine, D. J. , and T. L. Springer . 2013. “Competition and Facilitation Between a Native and a Domestic Herbivore: Trade‐Offs Between Forage Quantity and Quality.” Ecological Applications 23, no. 4: 850–863. 10.1890/12-0890.1. 23865235

[ece372102-bib-0007] Bänsch, S. , T. Tscharntke , R. Wünschiers , et al. 2020. “Using ITS2 Metabarcoding and Microscopy to Analyse Shifts in Pollen Diets of Honey Bees and Bumble Bees Along a Mass‐Flowering Crop Gradient.” Molecular Ecology 29, no. 24: 5003–5018. 10.1111/mec.15675. 33030785

[ece372102-bib-0008] Béland, S. , B. Vuillaume , M. Leclerc , M. Bernier , and S. D. Côté . 2023. “Selection of Summer Feeding Sites and Food Resources by Female Migratory Caribou ( *Rangifer tarandus* ) Determined Using Camera Collars.” PLoS One 18, no. 11: e0294846. 10.1371/journal.pone.0294846. 38019854 PMC10686509

[ece372102-bib-0009] Bergman, E. J. , P. F. Doherty , G. C. White , and A. A. Holland . 2015. “Density Dependence in Mule Deer: A Review of Evidence.” Wildlife Biology 21, no. 1: 18–29. 10.2981/wlb.00012.

[ece372102-bib-0010] Bolyen, E. , J. R. Rideout , M. R. Dillon , et al. 2019. “Reproducible, Interactive, Scalable and Extensible Microbiome Data Science Using QIIME 2.” Nature Biotechnology 37, no. 8: 852–857. 10.1038/s41587-019-0209-9. PMC7015180 31341288

[ece372102-bib-0011] Braukmann, T. W. A. , M. L. Kuzmina , J. Sills , E. V. Zakharov , and P. D. N. Hebert . 2017. “Testing the Efficacy of DNA Barcodes for Identifying the Vascular Plants of Canada.” PLoS One 12, no. 1: e0169515. 10.1371/journal.pone.0169515. 28072819 PMC5224991

[ece372102-bib-0012] Callahan, B. J. , P. J. McMurdie , M. J. Rosen , A. W. Han , A. J. A. Johnson , and S. P. Holmes . 2016. “DADA2: High‐Resolution Sample Inference From Illumina Amplicon Data.” Nature Methods 13, no. 7: 581–583. 10.1038/nmeth.3869. 27214047 PMC4927377

[ece372102-bib-0013] Castle, S. T. , N. Allan , D. Clifford , et al. 2020. “Diet Composition Analysis Provides New Management Insights for a Highly Specialized Endangered Small Mammal.” PLoS One 15, no. 10: e0240136. 10.1371/journal.pone.0240136. 33007017 PMC7531790

[ece372102-bib-0014] CBOL Plant Working Group , P. M. Hollingsworth , L. L. Forrest , et al. 2009. “A DNA Barcode for Land Plants.” Proceedings of the National Academy of Sciences of the United States of America 106, no. 31: 12794–12797. 10.1073/pnas.0905845106. 19666622 PMC2722355

[ece372102-bib-0015] Chase, M. W. , N. Salamin , M. Wilkinson , et al. 2005. “Land Plants and DNA Barcodes: Short‐Term and Long‐Term Goals.” Philosophical Transactions of the Royal Society, B: Biological Sciences 360, no. 1462: 1889–1895. 10.1098/rstb.2005.1720. PMC1609218 16214746

[ece372102-bib-0016] Chen, S. , H. Yao , J. Han , et al. 2010. “Validation of the ITS2 Region as a Novel DNA Barcode for Identifying Medicinal Plant Species.” PLoS One 5, no. 1: e8613. 10.1371/journal.pone.0008613. 20062805 PMC2799520

[ece372102-bib-0017] Cheng, T. , C. Xu , L. Lei , C. Li , Y. Zhang , and S. Zhou . 2016. “Barcoding the Kingdom Plantae: New pcr Primers for * its * Regions of Plants With Improved Universality and Specificity.” Molecular Ecology Resources 16, no. 1: 138–149. 10.1111/1755-0998.12438. 26084789

[ece372102-bib-0018] Chesson, P. 2000. “Mechanisms of Maintenance of Species Diversity.” Annual Review of Ecology and Systematics 31, no. 1: 343–366. 10.1146/annurev.ecolsys.31.1.343.

[ece372102-bib-0019] China Plant BOL Group , D.‐Z. Li , L.‐M. Gao , et al. 2011. “Comparative Analysis of a Large Dataset Indicates That Internal Transcribed Spacer (ITS) Should Be Incorporated Into the Core Barcode for Seed Plants.” Proceedings of the National Academy of Sciences of the United States of America 108, no. 49: 19641–19646. 10.1073/pnas.1104551108. 22100737 PMC3241788

[ece372102-bib-0020] Da Silva, L. P. , V. A. Mata , P. B. Lopes , et al. 2019. “Advancing the Integration of Multi‐Marker Metabarcoding Data in Dietary Analysis of Trophic Generalists.” Molecular Ecology Resources 19, no. 6: 1420–1432. 10.1111/1755-0998.13060. 31332947 PMC6899665

[ece372102-bib-0021] Deagle, B. E. , S. N. Jarman , E. Coissac , F. Pompanon , and P. Taberlet . 2014. “DNA Metabarcoding and the Cytochrome *c* Oxidase Subunit I Marker: Not a Perfect Match.” Biology Letters 10, no. 9: 20140562. 10.1098/rsbl.2014.0562. 25209199 PMC4190964

[ece372102-bib-0022] Durham, R. A. , D. L. Mummey , L. Shreading , and P. W. Ramsey . 2017. “Phenological Patterns Differ Between Exotic and Native Plants: Field Observations From the Sapphire Mountains, Montana.” Natural Areas Journal 37, no. 3: 361–381. 10.3375/043.037.0310.

[ece372102-bib-0023] Elbrecht, V. , and F. Leese . 2015. “Can DNA‐Based Ecosystem Assessments Quantify Species Abundance? Testing Primer Bias and Biomass—Sequence Relationships With an Innovative Metabarcoding Protocol.” PLoS One 10, no. 7: e0130324. 10.1371/journal.pone.0130324. 26154168 PMC4496048

[ece372102-bib-0024] Espinosa Prieto, A. , L. Hardion , N. Debortoli , and J. Beisel . 2024. “Finding the Perfect Pairs: A Matchmaking of Plant Markers and Primers for Multi‐Marker eDNA Metabarcoding.” Molecular Ecology Resources 24, no. 4: e13937. 10.1111/1755-0998.13937. 38363053

[ece372102-bib-0025] Fahner, N. A. , S. Shokralla , D. J. Baird , and M. Hajibabaei . 2016. “Large‐Scale Monitoring of Plants Through Environmental DNA Metabarcoding of Soil: Recovery, Resolution, and Annotation of Four DNA Markers.” PLoS One 11, no. 6: e0157505. 10.1371/journal.pone.0157505. 27310720 PMC4911152

[ece372102-bib-0026] Fonseca, V. G. 2018. “Pitfalls in Relative Abundance Estimation Using eDNA Metabarcoding.” Molecular Ecology Resources 18, no. 5: 923–926. 10.1111/1755-0998.12902.

[ece372102-bib-0027] Freeland, J. R. 2017. “The Importance of Molecular Markers and Primer Design When Characterizing Biodiversity From Environmental DNA.” Genome 60, no. 4: 358–374. 10.1139/gen-2016-0100. 28177833

[ece372102-bib-0028] Frøslev, T. G. , R. Kjøller , H. H. Bruun , et al. 2017. “Algorithm for Post‐Clustering Curation of DNA Amplicon Data Yields Reliable Biodiversity Estimates.” Nature Communications 8, no. 1: 1188. 10.1038/s41467-017-01312-x. PMC5662604 29084957

[ece372102-bib-0029] Hollingsworth, P. M. , S. W. Graham , and D. P. Little . 2011. “Choosing and Using a Plant DNA Barcode.” PLoS One 6, no. 5: e19254. 10.1371/journal.pone.0019254. 21637336 PMC3102656

[ece372102-bib-0030] Johnson, H. E. , G. L. Coulombe , L. G. Adams , et al. 2025. “DNA Metabarcoding and Video Camera Collars Yield Different Inferences About the Summer Diet of an Arctic Ungulate.” Ecosphere 16, no. 7: e70319. 10.1002/ecs2.70319.

[ece372102-bib-0031] Kartzinel, T. R. , P. A. Chen , T. C. Coverdale , et al. 2015. “DNA Metabarcoding Illuminates Dietary Niche Partitioning by African Large Herbivores.” Proceedings of the National Academy of Sciences 112, no. 26: 8019–8024. 10.1073/pnas.1503283112. PMC4491742 26034267

[ece372102-bib-0032] Khansaritoreh, E. , Y. Salmaki , E. Ramezani , et al. 2020. “Employing DNA Metabarcoding to Determine the Geographical Origin of Honey.” Heliyon 6, no. 11: e05596. 10.1016/j.heliyon.2020.e05596. 33294716 PMC7701183

[ece372102-bib-0033] King, S. R. B. , and K. A. Schoenecker . 2019. “Comparison of Methods to Examine Diet of Feral Horses From Noninvasively Collected Fecal Samples.” Rangeland Ecology & Management 72, no. 4: 661–666. 10.1016/j.rama.2019.02.005.

[ece372102-bib-0034] Kolter, A. , and B. Gemeinholzer . 2021a. “Internal Transcribed Spacer Primer Evaluation for Vascular Plant Metabarcoding.” Metabarcoding and Metagenomics 5: e68155. 10.3897/mbmg.5.68155.

[ece372102-bib-0035] Kolter, A. , and B. Gemeinholzer . 2021b. “Plant DNA Barcoding Necessitates Marker‐Specific Efforts to Establish More Comprehensive Reference Databases.” Genome 64, no. 3: 265–298. 10.1139/gen-2019-0198. 32649839

[ece372102-bib-0036] Krehenwinkel, H. , M. Wolf , J. Y. Lim , A. J. Rominger , W. B. Simison , and R. G. Gillespie . 2017. “Estimating and Mitigating Amplification Bias in Qualitative and Quantitative Arthropod Metabarcoding.” Scientific Reports 7, no. 1: 17668. 10.1038/s41598-017-17333-x. 29247210 PMC5732254

[ece372102-bib-0037] Lim, V.‐C. , R. Ramli , S. Bhassu , and J.‐J. Wilson . 2018. “Pollination Implications of the Diverse Diet of Tropical Nectar‐Feeding Bats Roosting in an Urban Cave.” PeerJ 6: e4572. 10.7717/peerj.4572. 29607265 PMC5875395

[ece372102-bib-0038] Liu, M. , C. P. Burridge , L. J. Clarke , S. C. Baker , and G. J. Jordan . 2023. “Does Phylogeny Explain Bias in Quantitative DNA Metabarcoding?” Metabarcoding and Metagenomics 7: e101266. 10.3897/mbmg.7.101266.

[ece372102-bib-0039] Mallona, I. , J. Weiss , and M. Egea‐Cortines . 2011. “pcrEfficiency: A Web Tool for PCR Amplification Efficiency Prediction.” BMC Bioinformatics 12, no. 1: 404. 10.1186/1471-2105-12-404. 22014212 PMC3234296

[ece372102-bib-0040] Martin, M. 2011. “Cutadapt Removes Adapter Sequences From High‐Throughput Sequencing Reads.” EMBnet.Journal 17, no. 1: 10. 10.14806/ej.17.1.200.

[ece372102-bib-0041] Meyer, J. M. , K. Leempoel , G. Losapio , and E. A. Hadly . 2020. “Molecular Ecological Network Analyses: An Effective Conservation Tool for the Assessment of Biodiversity, Trophic Interactions, and Community Structure.” Frontiers in Ecology and Evolution 8: 588430. 10.3389/fevo.2020.588430.

[ece372102-bib-0042] Moinard, S. , D. Piau , F. Laporte , et al. 2023. “Towards Quantitative DNA Metabarcoding: A Method to Overcome PCR Amplification Bias.” 10.1101/2023.10.03.560640.

[ece372102-bib-0043] Moorhouse‐Gann, R. J. , J. C. Dunn , N. De Vere , et al. 2018. “New Universal ITS2 Primers for High‐Resolution Herbivory Analyses Using DNA Metabarcoding in Both Tropical and Temperate Zones.” Scientific Reports 8, no. 1: 8542. 10.1038/s41598-018-26648-2. 29867115 PMC5986805

[ece372102-bib-0044] Moorhouse‐Gann, R. J. , I. P. Vaughan , N. C. Cole , et al. 2022. “Impacts of Herbivory by Ecological Replacements on an Island Ecosystem.” Journal of Applied Ecology 59, no. 9: 2245–2261. 10.1111/1365-2664.14096.

[ece372102-bib-0045] Newmaster, S. G. , I. D. Thompson , R. A. D. Steeves , et al. 2013. “Examination of Two New Technologies to Assess the Diet of Woodland Caribou: Video Recorders Attached to Collars and DNA Barcoding.” Canadian Journal of Forest Research 43, no. 10: 897–900. 10.1139/cjfr-2013-0108.

[ece372102-bib-0046] Nichols, R. V. , C. Vollmers , L. A. Newsom , et al. 2018. “Minimizing Polymerase Biases in Metabarcoding.” Molecular Ecology Resources 18, no. 5: 927–939. 10.1111/1755-0998.12895. 29797549

[ece372102-bib-0047] Oksanen, J. , G. L. Simpson , F. G. Blanchet , et al. 2024. “vegan: Community Ecology Package.” https://CRAN.R‐project.org/package=vegan.

[ece372102-bib-0048] Padmalal, U. K. G. K. , S. Takatsuki , and P. Jayasekara . 2003. “Food Habits of Sambar *Cervus unicolor* at the Horton Plains National Park, Sri Lanka.” Ecological Research 18, no. 6: 775–782. 10.1111/j.1440-1703.2003.00595.x.

[ece372102-bib-0049] Pansu, J. , M. C. Hutchinson , T. M. Anderson , et al. 2022. “The Generality of Cryptic Dietary Niche Differences in Diverse Large‐Herbivore Assemblages.” Proceedings of the National Academy of Sciences of the United States of America 119, no. 35: e2204400119. 10.1073/pnas.2204400119. 35994662 PMC9436339

[ece372102-bib-0050] Pedregosa, F. , G. Varoquaux , A. Gramfort , et al. 2011. “Scikit‐Learn: Machine Learning in Python.” Journal of Machine Learning Research 12, no. Oct: 2825–2830.

[ece372102-bib-0051] Piñol, J. , M. A. Senar , and W. O. C. Symondson . 2019. “The Choice of Universal Primers and the Characteristics of the Species Mixture Determine When dna Metabarcoding Can Be Quantitative.” Molecular Ecology 28, no. 2: 407–419. 10.1111/mec.14776. 29939447

[ece372102-bib-0052] Pitteloud, C. , E. Defossez , C. Albouy , P. Descombes , S. Rasmann , and L. Pellissier . 2023. “ dna‐Based Networks Reveal the Ecological Determinants of Plant–Herbivore Interactions Along Environmental Gradients.” Molecular Ecology 32, no. 23: 6436–6448. 10.1111/mec.16545. 35620937

[ece372102-bib-0053] Pringle, R. M. , J. O. Abraham , T. M. Anderson , et al. 2023. “Impacts of Large Herbivores on Terrestrial Ecosystems.” Current Biology 33, no. 11: R584–R610. 10.1016/j.cub.2023.04.024. 37279691

[ece372102-bib-0054] Quin, M. J. , J. W. Morgan , and N. P. Murphy . 2023. “Spatial and Temporal Variation in the Diet of Introduced Sambar Deer (*Cervus unicolor*) in an Alpine Landscape.” Wildlife Research 51, no. 1: WR23017. 10.1071/WR23017.

[ece372102-bib-0055] R Core Team . 2021. R: A Language and Environment for Statistical Computing. R Foundation for Statistical Computing. https://www.R‐project.org/.

[ece372102-bib-0056] Rieseberg, L. H. , T. E. Wood , and E. J. Baack . 2006. “The Nature of Plant Species.” Nature 440, no. 7083: 524–527. 10.1038/nature04402. 16554818 PMC2443815

[ece372102-bib-0057] Rognes, T. , T. Flouri , B. Nichols , C. Quince , and F. Mahé . 2016. “VSEARCH: A Versatile Open Source Tool for Metagenomics.” PeerJ 4: e2584. 10.7717/peerj.2584. 27781170 PMC5075697

[ece372102-bib-0058] Scasta, J. D. , D. L. Lalman , and L. Henderson . 2016. “Drought Mitigation for Grazing Operations: Matching the Animal to the Environment.” Rangelands 38, no. 4: 204–210. 10.1016/j.rala.2016.06.006.

[ece372102-bib-0059] Sickel, W. , M. J. Ankenbrand , G. Grimmer , et al. 2015. “Increased Efficiency in Identifying Mixed Pollen Samples by Meta‐Barcoding With a Dual‐Indexing Approach.” BMC Ecology 15, no. 1: 20. 10.1186/s12898-015-0051-y. 26194794 PMC4509727

[ece372102-bib-0060] Simberloff, D. , and T. Dayan . 1991. “The Guild Concept and the Structure of Ecological Communities.” Annual Review of Ecology and Systematics 22: 115–143.

[ece372102-bib-0061] Stadhouders, R. , S. D. Pas , J. Anber , J. Voermans , T. H. M. Mes , and M. Schutten . 2010. “The Effect of Primer‐Template Mismatches on the Detection and Quantification of Nucleic Acids Using the 5′ Nuclease Assay.” Journal of Molecular Diagnostics 12, no. 1: 109–117. 10.2353/jmoldx.2010.090035. PMC2797725 19948821

[ece372102-bib-0062] Stafford, K. J. 1997. “The Diet and Trace Element Status of Sambar Deer (*Cervus unicoloi*) in Manawatu District, New Zealand.” New Zealand Journal of Zoology 24, no. 4: 267–271. 10.1080/03014223.1997.9518123.

[ece372102-bib-0063] Stewart, K. M. , R. T. Bowyer , J. G. Kie , and M. A. Hurley . 2010. “Spatial Distributions of Mule Deer and North American Elk: Resource Partitioning in a Sage‐Steppe Environment.” American Midland Naturalist 163, no. 2: 400–412. 10.1674/0003-0031-163.2.400.

[ece372102-bib-0064] Stoner, D. C. , M. T. Anderson , C. A. Schroeder , C. A. Bleke , and E. T. Thacker . 2021. “Distribution of Competition Potential Between Native Ungulates and Free‐Roaming Equids on Western Rangelands.” Journal of Wildlife Management 85, no. 6: 1062–1073. 10.1002/jwmg.21993.

[ece372102-bib-0065] Swenson, S. J. , and B. Gemeinholzer . 2021. “Testing the Effect of Pollen Exine Rupture on Metabarcoding With Illumina Sequencing.” PLoS One 16, no. 2: e0245611. 10.1371/journal.pone.0245611. 33529182 PMC7853484

[ece372102-bib-0066] Taberlet, P. , E. Coissac , F. Pompanon , et al. 2007. “Power and Limitations of the Chloroplast trnL (UAA) Intron for Plant DNA Barcoding.” Nucleic Acids Research 35, no. 3: e14. 10.1093/nar/gkl938. 17169982 PMC1807943

[ece372102-bib-0067] Thomassen, E. E. , E. E. Sigsgaard , M. R. Jensen , et al. 2023. “Contrasting Seasonal Patterns in Diet and Dung‐Associated Invertebrates of Feral Cattle and Horses in a Rewilding Area.” Molecular Ecology 32, no. 8: 2071–2091. 10.1111/mec.16847. 36744391

[ece372102-bib-0068] Thompson, I. D. , M. Bakhtiari , A. R. Rodgers , J. A. Baker , J. M. Fryxell , and E. Iwachewski . 2012. “Application of a High‐Resolution Animal‐Borne Remote Video Camera With Global Positioning for Wildlife Study: Observations on the Secret Lives of Woodland Caribou.” Wildlife Society Bulletin 36, no. 2: 365–370. 10.1002/wsb.130.

[ece372102-bib-0069] Timpano, E. K. , M. K. R. Scheible , and K. A. Meiklejohn . 2020. “Optimization of the Second Internal Transcribed Spacer (ITS2) for Characterizing Land Plants From Soil.” PLoS One 15, no. 4: e0231436. 10.1371/journal.pone.0231436. 32298321 PMC7162488

[ece372102-bib-0070] White, T. J. , T. D. Bruns , S. B. Lee , and J. W. Taylor . 1990. Amplification and Direct Sequencing of Fungal Ribosomal RNA Genes for Phylogenetics. Academic Press.

[ece372102-bib-0071] Wickham, H. 2016. ggplot2: Elegant Graphics for Data Analysis. Springer‐Verlag. https://ggplot2.tidyverse.org.

[ece372102-bib-0072] Wilson, R. S. , A. Keller , A. Shapcott , et al. 2021. “Many Small Rather Than Few Large Sources Identified in Long‐Term Bee Pollen Diets in Agroecosystems.” Agriculture, Ecosystems & Environment 310: 107296. 10.1016/j.agee.2020.107296.

[ece372102-bib-0073] Yamamoto, S. , and K. Uchida . 2018. “A Generalist Herbivore Requires a Wide Array of Plant Species to Maintain Its Populations.” Biological Conservation 228: 167–174. 10.1016/j.biocon.2018.10.018.

[ece372102-bib-0074] Yao, H. , J. Song , C. Liu , et al. 2010. “Use of ITS2 Region as the Universal DNA Barcode for Plants and Animals.” PLoS One 5, no. 10: e13102. 10.1371/journal.pone.0013102. 20957043 PMC2948509

